# Long-Term Outcomes of Liver Transplantation for the Management of Neuroendocrine Neoplasms: A Systematic Review

**DOI:** 10.3390/jpm13101428

**Published:** 2023-09-23

**Authors:** Varun Palaniappan, Chun Hei Li, Andrea Frilling, Ashley Kieran Clift

**Affiliations:** 1 Cardiff and Vale University Health Board, Cardiff CF14 4XW, UK; 2 St George’s University Hospitals NHS Foundation Trust, London SW17 0QT, UK; 3 Department of Surgery & Cancer, Imperial College London, London SW7 2BX, UK; 4 Cancer Research UK Oxford Centre, University of Oxford, Oxford OX1 2JD, UK

**Keywords:** neuroendocrine, transplantation, outcomes, liver metastases, systematic review

## Abstract

Liver transplantation is an uncommonly used, controversially debated therapeutic approach for highly selected individuals with neuroendocrine liver metastases. Synthesising evidence regarding outcomes from this approach is crucial to understand its position within the broad neuroendocrine liver metastases armamentarium. In this narrative systematic review of studies published in PubMed, Scopus and OVID until 1 July 2021, we summarise and critically appraise the existing literature regarding this modality, with a special focus on long-term outcomes data where possible. Fourteen studies were identified that reported outcomes from the use of liver transplantation for metastatic neuroendocrine neoplasms. No randomised trials were identified. Generally, indications and selection criteria were poorly articulated, with the notable exception of studies using the Milan criteria. The median 5-year overall survival was 65% (ranging from 36% to 97.2%, 11 studies), and the median 10-year overall survival was 50% (ranging from 46.1% to 88.8%, 3 studies). One additional study focussed on treatments and outcomes following post-transplant recurrence. No studies reported outcomes past 10 years. Further follow-up of the largest series with explicit selection criteria will deepen our understanding of the role that transplantation has to play in this setting.

## 1. Introduction

Neuroendocrine neoplasms (NEN) are an increasingly prevalent class of tumour that arise from multiple organs, but most commonly in the gastroenteropancreatic tract, or bronchopulmonary system [1]. Clinical challenges include the symptomatic burdens of hormonally active (“functional”) tumours, their propensity to metastasise despite their generally accepted relative indolent growth, and high incidence of nodal and/or distant metastases at initial presentation [2]. Specialist centre experience documents that up to 90% of small intestinal NEN display evidence of nodal metastases at diagnosis [3], with up to 91% of small intestinal NEN patients and up to 77% of pancreatic NEN patients developing hepatic metastases [4,5,6]. Gold-standard imaging also significantly understages metastatic disease in the liver [7], mandating careful treatment strategy and according follow-up. Whilst patients harbouring neuroendocrine liver metastases (NELM) may have protracted survival, particularly when compared to expected prognosis of stage IV gastrointestinal adenocarcinomas, the presence of NELM exerts a major, negative prognostic effect, and multimodal treatment is often required to attain disease control [2,8].

Recent advances, driven by increased centralisation of care into expert centres/networks and inter-centre collaboration, have expanded the therapeutic armoury, such as evidence from randomised controlled trials supporting the use of somatostatin analogues (SSAs) [9], peptide receptor radionuclide therapy (PRRT) [10], molecularly targeted agents [11,12], and interventional “trans-arterial” procedures such as selective internal radioembolisation for hepatic disease [13]. However, whilst initial reports were promising regarding the anti-proliferative effects of SSAs and PRRT in terms of progression-free survival, there is no evidence that they significantly affect overall survival [14].

Radical surgical intervention is therefore the only modality that possesses the opportunity to attain cure—this is possible in the setting of NELM, with the resection of locoregional tumour burden and liver metastases if oncologically and technically appropriate [15]. A minority of patients with NELM are candidates for resection with curative intent, and even if R0 margins are attained, disease recurrence is a significant hindrance [16], leading some to posit that for many cases, “curative resection” is a palliative endeavour albeit with a sustained duration of disease control [15]. The other aspect of the surgical armamentarium for NELM [17] or advanced primary hepatic NEN [18,19] is liver transplantation, either in the classic orthotopic fashion, or as part of a multivisceral graft [20,21,22,23]. Indications for OLT generally comprise control of medically intractable symptoms from functional tumours, amelioration of effects of hepatic tumour bulk, or for oncological control [24]. Initial outcomes with OLT for NELM were very poor [17,24]; however, technical progress and implementation of stringent selection criteria have been shown to be associated with improved prognosis [25,26,27]. Other centres have demonstrated excellent results exceeding those seen for liver resection based on meticulous selection criteria [28], and some reports have demonstrated the possibility of excellent long-term survival. However, given the rarity of the indication (<1% of all liver transplant activity) [22], summarising the available evidence into a cohesive platform is necessary to understand the divergences in practice and outcomes that generate debate around this contested modality. Specifically, there is lack of robust data on long-term survival outcomes, e.g., 10 years or longer.

Here, we undertake a systematic review of the literature regarding the use of liver transplantation for the treatment of NEN, seeking to update the findings of a previous review [17], extend prior work by critically appraising the evidence limitations and outline avenues for further study. This is done with a specific focus on long-term outcomes of this approach.

## 2. Materials and Methods

### 2.1. Protocol Registration and Study Conduct

The protocol for this systematic review was registered on the PROSPERO database (reference CRD42021267963; https://www.crd.york.ac.uk/prospero/display_record.php?ID=CRD42021267963, accessed on 1 August 2021) prior to the commencement of the study. The systematic review was undertaken cognisant of and reported according to the PRISMA guidelines [29] (checklist in Supplementary File S1).

We formulated our review considering the “PICO” framework:

Population: patients with advanced neuroendocrine neoplasms (liver metastases);
Intervention: liver transplantation, either alone (orthotopic) or part of a multivisceral graft;
Comparison: other, non-transplant treatment strategies (data availability permitting);
Outcome: overall and disease-free survival at 1-, 3-, 5-, 10-, and 10+ years.

### 2.2. Search Strategy, Data Sources and Inclusion

We undertook a systematic review of three databases: PubMed (MEDLINE), Scopus Web of Science and EMBASE. Search terms focussed on “neuroendocrine” and “liver and “transplant”—the full search strings are available via the link on the registered protocol webpage on PROSPERO (https://www.crd.york.ac.uk/PROSPEROFILES/267963_STRATEGY_20210714.pdf, accessed on 1 August 2021) and in Supplementary File S2. There were no amendments to the protocol.

Papers published until 1 July 2021 written in English or German were considered for inclusion. We considered publications reporting on liver transplantation for NEN or NELM, whether or not they were purely NEN cohorts (provided that NEN-specific data were reported), or reports discussing several primary tumour types. Papers were excluded if they were animal studies, review articles, another systematic review with or without meta-analysis, or editorials or other non-research studies not reporting outcomes data. Case reports were not included for data extraction. If data from the same centre or registry were presented in multiple reports, we selected the most recently published paper.

Three authors each performed the search string on one source (VP EMBASE, DL on PubMed, AKC Scopus). The number of articles was noted, and all records uploaded onto the Rayyan platform for duplicate identification and screening. Three independent reviewers (VP, DL and AKC) screened all articles (titles and abstracts), with each record assessed twice. Conflicts were resolved in group teleconference. AKC also reviewed reference lists of the finally selected papers for additional potential references.

### 2.3. Data Extraction and Summary

All selected articles passing title and abstract screening were assessed by two independent reviewers for potential data extraction: VP and DL reviewed 50% each of all articles, with AKC performing the second review. Disagreements were discussed in group teleconference. A pre-developed data extraction template was trialled using two large, previously known studies to assess appropriateness and ease of use.

Thereafter, data were extracted regarding first author, year of publication, country, study design, number of patients included with NEN, median age of participants (or other summary statistics if presented), male:female numbers, primary tumour types, tumour histology, median follow-up (or other summary statistic), overall survival at 1, 3, 5, 10, and 10+ years, recurrence-free survival at 1, 3, 5, 10, and 10+ years, type of immunosuppression, pre-transplant treatment information, and “miscellaneous” data felt to be of relevance by individual reviewers.

Per-study rows were permitted to be split if the study authors reported outcomes for distinct temporal periods, or if they reported outcomes for specific forms of transplantation (e.g., orthotopic liver transplantation, or multivisceral transplantation). Outcome data were extracted “as is”—in cases where no Kaplan–Meier/other actuarial survival metrics were reported, we did not compute by hand using raw data (if provided).

### 2.4. Synthesis and Meta-Analysis

The findings of this systematic review were synthesised narratively. We had considered pooling of identified Kaplan–Meier estimates using random effects meta-analysis. However, due to the high heterogeneity in centre selection criteria and follow-up duration, and notable trends in outcomes over time, we felt that this was inappropriate and would lead to imprecise estimates. As the included estimates were overwhelmingly case series, we used the Institute for Health Economics (Edmonton, AB, Canada) quality appraisal checklist: (https://www.ihe.ca/download/development_of_a_quality_appraisal_tool_for_case_series_studies_using_a_modified_delphi_technique.pdf, accessed on 1 August 2021).

## 3. Results

**Figure 1:**
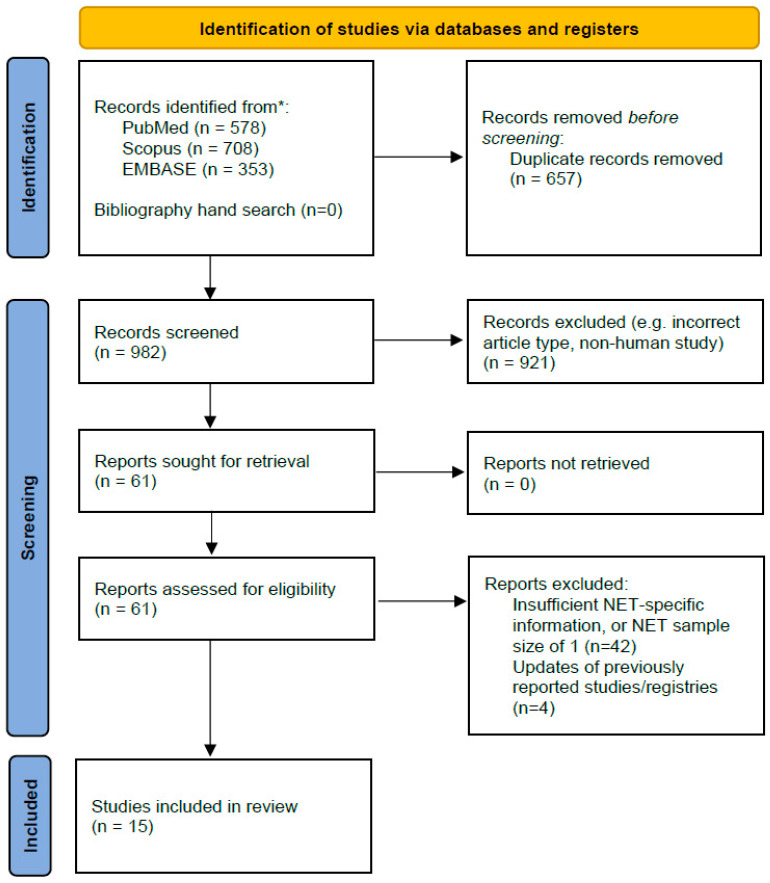
PRISMA flowchart describing the steps in the systematic review from literature search to included studies. * = distinct record databases.

The reference selection process is illustrated in the PRISMA flowchart in Figure 1. From the initially extracted 982 records, we included 15 studies in the final evidence synthesis: 14 studies reporting survival outcomes after LT for NEN, and 1 reporting on outcomes after post-OLT recurrence. These studies are presented in Table 1. Four papers that reported relevant data were deemed to be older, preceding reports of the same/highly overlapping patient pools [25,30,31,32] and therefore not included in the final summary. As all records were retrospective case series, this review remained “narrative” and without meta-analysis. A relative quality appraisal is summarised in Supplementary File S3.

The publication date of the included studies ranged from 1995 to 2021, covering study periods starting in 1983 onwards. Sample sizes ranged between 5 [38] and 213 [24]. There was non-uniform reporting of median age of the cohorts, summary characteristics of follow-up, sex of participants, the survival outcomes reported (intervals and whether this was overall survival or disease-free), prior treatment strategies and selection criteria. Whilst most studies reported solely on outcomes following orthotopic liver transplantation for NELM [24], some included cases of primary hepatic NEN, and some included patients undergoing multivisceral transplantation for advanced tumours including hepatic metastases [20,23,40]. Interestingly, the recent case series of Sposito et al. reported outcomes following recurrence after OLT [43].

Generally, selection criteria for OLT (or MVT) were poorly described, although some studies provided a breakdown of indications, e.g., for intractable symptoms [24]. The exception was the experience of the Mazzaferro et al. with the modified Milan NET criteria [28], which are discussed below.

Across studies, median overall survival at 1 year was 87% (range 73%–100%, 11 studies), and at 5 years this was 65% (range 36%–97.2%, 11 studies). Only four studies [28,34,42,43] reported overall survival at 10 years—two of these had overlapping patient cohorts [28,43], so after retaining the source/main study [28] (the other focussed on post-OLT-recurrence outcomes, rather than outcomes post OLT in general), the median 10 year OS was 50%, ranging from 46.1% to 88.8% (three studies). None reported overall survival outcomes past 10 years. Overall, the overall survival data appeared more optimistic than those reported for the accompanying disease-free/recurrence-free survival: at 1 year, this was 70% (range 56%–80%, seven studies), and at 5 years, this was 36.5% (range 11%–86.9%, eight studies). Similarly, only two studies reported 10-year disease-free survival which was wide ranging: 21% [34] and 86.9% [28]. None reported outcomes after 10 years. Due to the profound heterogeneity in selection criteria, time periods, temporal trends in outcomes over time [42] (e.g., with implementation of scoring systems/organ allocation methods), a random effects meta-analysis was not performed due to the low likelihood of providing meaningful pooled estimate that translates into current practice. Furthermore, if such a meta-analysis was feasible, a meta-regression to examine factors associated with the expected high degree of heterogeneity would be infeasible due to poor recording of potentially attributable factors.

The largest study identified was the multicentric report of the European Liver Transplant Registry, which evaluated outcomes in 213 patients with NEN metastatic to the liver between 1982 and 2009 [24]. The treatment of disease prior to LT included resection of the primary tumour and/or hepatic deposits in over 80% of cases, and a high use of prior chemotherapy (71%, including systemic or intra-arterial liver-directed modalities). Indications for LT were for oncological control (54%), to treat the effects of tumoral bulk (24%, presumably predominantly hepatic burden leading to pain), and control of hormonal excess/functional syndrome (17%). Alongside reporting long-term outcomes (e.g., 5-year OS 52%, 5-year DFS 30%), the authors noted improved longer-term overall survival in the most recent temporal period (5-year OS 59% after 2000, n = 106, compared to 46% prior to 2000). Le Treut and colleagues undertook an evaluation of prognostic factors using data for the 106 patients treated after 2000, and generated a simple 4-point score based thereon. This score incorporated three baseline/pre-treatment information factors: hepatomegaly, age over 45 years, and concomitant additional resection, with prognostic scores ranging between 0 and 3. Furthermore, the authors demonstrated significantly divergent overall survival curves when separated into two groups (0–1 factor versus 2–3 factors present, 79% OS versus 38%, respectively, and 5-year DFS 38% and 19%, respectively). However, whilst this prognostic score is attractive in its simplicity and apparent ability to stratify, it presents several methodological issues. These include the dichotomization of age (which risks information waste and step artefacts [44]), univariate screening of predictors [44], and that the tool is only evaluated in terms of separated survival curves, rather than being assessed in terms of calibration discrimination and clinical utility. Whilst the prognostic score was developed, and the indications for OLT reported, clear centre-specific selection criteria were not available [24].

Evidence from multi-centre series in the United States supports the evidence from European centres regarding improvement in long-term outcomes with LT for NELM over time. For example, in their analysis of the United States Organ Sharing/Organ Procurement and Transplantation Network (UNOS/OPTN) covering recorded transplants between 187 and 2011 (184 for NELM), Nguyen and colleagues [27] reported that survival significantly improved after introduction of the MELD/PELD score in 2002. Indeed, initially observed differences in long-term outcomes between transplants for NELM and transplants for hepatocellular carcinoma were negated to non-significance after this time point, with 5-year OS for NELM patients at 57.8%, versus 64.4% for HCC (*p* = 0.109) [27]. Iteratively updated reports using the UNOS/OPTN database have been published, such as those by Gedaly et al. [25], and most recently, Valvi et al. [42]. The latter study of 206 patients undergoing “isolated” liver transplantation for metastatic NEN (of a total 160,360 total liver transplants between 1988 and 2018) reported a 5-year OS of 64.9% and a 10-year OS of 46.1%, and explored the role of potential prognostic factors on risk of death or recurrence [42]. Propensity score matching (on MELD score and gender) was used to match NEN to HCC and cholangiocarcinoma patients at a ratio of 1:3 to perform comparisons of outcomes in these groups. The NELM group was observed to have a higher incidence of recurrence (34%) versus HCC (8%) or cholangiocarcinoma patients (19.6%), however there were no significant differences in overall survival between these groups. For example, 5-year survival for NELM was 75.4%, 79.9% for HCC, and 70.4% for the cholangiocarcinoma group. Furthermore, an effect of duration on transplant waiting list was observed for recurrence in NEN patients—in those that recurred after liver transplantation, 74.3% waited for under 6 months, whereas 25.7% were on the waiting list for longer than 6 months. Limitations of propensity score matching include reduced sample size, counterintuitive risk of imbalance, dependence on the matching model, and the fact that it does not eliminate confounding.

The only study to meticulously define and adhere to a single set of selection criteria is that of Mazzaferro et al. who documented their prospective experience with patient selection according to their modified Milan criteria for NELM [28]. These criteria were set in 1995 and comprise confirmed histology of G1 or G2 NEN, primary tumour drained by the hepatic portal system and removed as well as extra-hepatic deposits in a separate curative resection prior to consideration for OLT, <50% total liver involvement, at least 6 months of stable disease/disease response prior to consideration of OLT and age < 60 years (relative criterion). Eighty-eight patients with NELM eligible for OLT were included, with two sub-groups analysed: those that underwent transplantation (n = 42), and those that did not (n = 46; 22 refusals/non-compliant, 24 due to transplant list unavailability). Regarding follow-up imaging strategy, 3–4 monthly CT or MRI imaging was used, with somatostatin receptor-based imaging (OctreoScan or 68-Gallium PET/CT) only used in cases of suspicion of recurrence on CT or MRI. Patients undergoing OLT had significantly smaller tumours, were younger (median age 40.5 years versus 55.5 years), and underwent more locoregional therapy (40.5% versus 21.7%) than those that did not receive a transplant. Statistical analyses incorporated adjustment for propensity scores (i.e., propensity to receive a transplant), the models for which incorporated a suite of clinicopathological characteristics and underwent robust derivation, assessment, and implementation. Excellent long-term outcomes were observed with OLT, with 97.2% and 88.8% OS at 5 and 10 years, respectively, compared to 50.9% and 22.4%, respectively in the non-transplant group. Post-transplant disease recurrence was very low, at 13.1% at 10 years. Furthermore, the survival benefits of OLT increased over time, with the adjusted benefit at 5-year follow-up estimated at 6.82 months (95% CI: 1.10 months to 12.54 months) and 38.43 months (95% CI: 21.41 months to 55.45 months). It has been posited that many of the patients undergoing OLT in this study may have been suitable candidates for liver resection, and naturally, these patients are highly selected. Nevertheless, the 86.9% DFS at 10 years with the Milan approach appears exemplary and is worthy of further robust consideration.

The same group reported their experience managing patients that were selected for OLT, but developed post-LT recurrence (study period 2004 to 2018) [43]. This retrospective case series comprised 32 patients treated at the same centre as the previous discussed study [28], and thus, in this review, we focussed separately on their post-recurrence outcomes [43]. Follow-up imaging included OctreoScan or 68-Gallium PET/CT. Recurrence was most commonly at a single site (81.2% of cases), particularly in the distant lymph nodes (40.6%) or locoregional lymph nodes (18.8%), but this also manifested as peritoneal or pulmonary lesions. The inadequacy of chromogranin A for post-transplant surveillance was suggested, as only 12 patients (37.5%) had elevated levels at the time recurrence was ascertained. Fourteen patients (43.8%) underwent treatment with radical intent, with 13/14 having no evidence of disease at follow-up radiology at 3 months. Other individuals that were not candidates for aggressive recurrence treatment due to non-resectability received chemotherapy, PRRT or SSAs. Within a median follow-up from recurrence of 73.7 months, 5- and 10-year post-recurrence overall survival was estimated to be 76.3% and 45.5%, respectively, suggesting that, even in cases of post-LT recurrence, favourable long-term outcome is attainable. However, this must be considered in terms of the relatively small sample size at a single institution, as must their analyses of prognostic factors (not discussed here).

## 4. Discussion

Liver transplantation may be associated with favourable long-term outcomes in NET patients with advanced disease—for example, the median 10 year OS was 50%, range 46.1%–88.8%. Recurrence-free survival was inconsistently reported, with two studies (varying in veracity of patient selection) reporting 10-year DFSs of 21% and 86.9%.

Liver transplantation offers a seemingly attractive yet uncommonly used approach for the aggressive management of metastatic neuroendocrine liver metastases. Whilst hepatectomy with curative intent is associated with favourable outcomes in NELM, very high recurrence rates are a major limitation of this approach [15,16], likely driven by understaging of hepatic disease, which is observed even with gold-standard imaging with ensuing residual micro-disease burden [7]. Thus, aggressive treatment manifesting as total hepatectomy (i.e., complete removal of all macro- and micro- disease in the liver) with resection of primary and extrahepatic disease, and a liver allograft appears to be a legitimate option when one considers the excellent results reported by meticulously selected criteria.

Compared to the systematic review of Moris and colleagues [17] on liver transplantation for metastatic NEN, we identified updated multicentre experience from the United States (UNOS database) [42] reinforcing evidence that post-OLT outcomes have improved over time, and included the first study to report in detail on outcomes in cases of recurrence post-OLT [43], which suggests that even in these cases, multimodal therapy may be useful to prolong survival. Nevertheless, our overall conclusions align—documentation of selection criteria is generally poor to non-existent in most studies, there is inconsistent recording of indication, immunosuppression regimens, and patients tend to be heavily pre-treated [24,28]. While studies report 1-, 3-, 5-, and 10-year survival data, here is a lack of studies reporting outcomes after 10 years. Such data are essential in order to understand the true value of liver transplantation on disease outcomes.

There is some evidence that outcomes with OLT for NELM in the recent era in selected patients are not inferior to other, arguably more established “transplant oncology” indications [27,42]. However, the primary evidence gap limiting promotion of OLT pertains to selection—strict selection of patients is necessary, but the optimal approach is not discernible from current evidence.

In order to rectify this, robust derivation and evaluation of multivariable selection models in pooled, specialist centre data may offer utility. Movement away from reductionist, retrospectively derived “scores” with no solid validation towards a more nuanced risk estimation tool that is prospectively evaluated would represent a significant advance. As aforementioned, merely stratifying patients into two vague risk groups only demonstrates broad “average” expectancies, and the use of arbitrary cut-offs in such tools poses ethical questions that become rapidly apparent [24]. External implementation of the Milan NET criteria in other centres should be considered, with meticulous data collection, prospective validation and transparent reporting. Indeed, a novel transplant programme for NELM has been recently initiated for the United Kingdom and Ireland [45], which will be a valuable addition to the evidence base as they report later.

We conclude that, given the currently available evidence and limitations of this evidence, further single-centre or purely retrospective case series will not present any additional benefit to the literature. The relative rarity of NEN and uncommon consideration of NELM for OLT presents logistical challenges, but with centralisation of care and through international interest groups, multi-centre collaboration for such prospective studies is not only possible, but something that is actively being arranged [45]. The literature for transplantation in the management of neuroendocrine liver metastases needs to mature, and relevant stakeholders must drive this. As part of these new studies, novel protocols could include the consideration of adjuvant therapies to reduce the risk of disease recurrence, should incorporate gold standard imaging protocols as part of follow-up and could consider novel omics-based biomarker technologies [46,47] to expedite the diagnosis of recurrent disease.

## Figures and Tables

**Table 1:** Summary of included studies regarding liver transplantation for the management of neuroendocrine neoplasms. OS = overall survival, DFS = disease-free survival, OLT = orthotopic liver transplantation, MVT = multivisceral transplantation, NR = not reported.

First Author	Year of Publication	Study Period	Country/Ies	Study Design	Sample Size	Median Age	Gender (M:F)	Median Follow-Up	1-Year OS	3-Year OS	5-Year OS	10-Year OS	1-Year DFS	3-Year DFS	5-Year DFS	10-Year DFS
Routley [33]	1995	1983–1997	United Kingdom	Multicentre, retrospective case series	11	NR	6:5	NR	82%		57%					
Rosenau [34]	2002	1982–1997	Germany	Single centre, retrospective case series	19	Median 47 years (range 18–61)	9:10	Mean 59 months (0.5–146)	89%		80%	50%	56%		21%	21%
Florman [35]	2004	1992–2002	United States	Single centre, retrospective case series	11	Mean 51.2 +/− 6.3 yrs	4:7	Mean 34 +/− 40 months	73%		36%					
van Vilsteren [36]	2006	1998–2002	United States	Single centre, retrospective case series	17	Median 47 years (range 22–64)	15:4	Mean 22 months (range 0–84)	87%				77%			
Marin [37]	2007	1996–2006	Spain	Single centre, retrospective case series	10	Mean 42 years (range 30–62)	5:5	Mean 3 years, range 1 month–6 years	86%	57%						
Olausson [23]	2007	1997–2001	Sweden	Single centre, retrospective case series	15 (10 OLT, 5 MVT)	Median 51.5 years (range 39–64) OLT. Median 43 years (range 38–57) for MVT	11:4	Mean 53.8 months (+/−9.5)			90% OLT		Approx. 70% for all patients		20%	
Dhupar [38]	2009	1991–2006	United States	Single centre, retrospective case series	5	Median 44 years (range 17–53)	2:3	NR	100%							
Frilling [39]	2009	NR	Germany	Single centre, retrospective case series	17	NR	NR	NR			67%				48%	
Bonaccorsi-Riani [40]	2010	NR	Belgium	Single centre, retrospective case series	9	Median 54 years (range 26.6–61)	7:2	NR	88%	77%	33%		67%	33%	11%	
Le Treut [24]	2013	1982–2009	Multiple in Europe	Multicentre, retrospective case series	213	Mean 46 years +/− 11. Median 48 years (range 16–71)	114:99	Mean 56 +/− 49 months (range 0–283)	81%	65%	52%		65%	40%	30%	
Sher [20]	2015	1988–2012	United States, Canada, Europe	Multicentre, retrospective case series	85	Median 48 years (range 16–75)	51:34	Median 2.7 years (range 0.05–21.4)	83%	60%	52%					
Mazzaferro [28]	2016	1995 onwards	Italy	Single centre, retrospective case series	42	Median 40.5 (range 13–62)	26:16	NR			97.2%	88.8%			86.9%	86.9%
Korda [41]	2019	1995–2018	Hungary	Single centre, retrospective case series	10	Median 49.5 years (range 38–62)	4:6	Median 33 months (range 9–104)	89%		71%		80%		43%	
Valvi [42]	2021	1988–2018	United States	Multicentre, retrospective case series	206	Mean 48.2 years (SD 11.7, range 19–75)	117:89	NR	89%	75.3%	65%	46.1%	74.9%	55.7%	43.9%	
Post-OLT recurrence																
Sposito [43]	2021	2004–2018	Italy	Single centre, retrospective case series	53 had LT; 32 recurred	At recurrence, median 55 (range 48.5–60.3)	16:15	Median 73.7 months after recurrence			76.3%	45.5%				

## Supplementary Materials

The following supporting information can be downloaded at: https://www.mdpi.com/article/10.3390/jpm13101428/s1, Supplementary File S1: PRISMA checklist, Supplementary File S2: Search strategy; Supplementary File S3: Quality appraisal.
