# Supplementary material for: Long-Term Outcomes of Liver Transplantation for the Management of Neuroendocrine Neoplasms: A Systematic Review
Source: J Pers Med. 2023 Sep 23;13(10):1428. doi: 10.3390/jpm13101428 (PMC10607978; doi:10.3390/jpm13101428)
Supplement: Supplementary file 1 [file jpm-13-01428-s001.zip › File S1_Searchstrategy.pdf]

PubMed: "neuroendocrine"[Title/Abstract] AND "liver" AND "transplant*"

Scopus: TITLE-ABS-KEY ("neuroendocrine" AND "liver" AND "transplant*")

EMBASE:
1. neuroendocrine tumor/ or neuroendocrine tumo*.mp or carcinoid/
2. liver transplantation.mp. or liver transplantation/
3. liver transplant.mp. or liver graft/
4. hepatic transplantation.mp.
5. liver metastasiss/ or liver metastas*.mp.
6. 2 or 3 or 4
7. 1 and 6
8. limit 8 to human
