# Supplementary material for: Long-Term Outcomes of Liver Transplantation for the Management of Neuroendocrine Neoplasms: A Systematic Review
Source: J Pers Med. 2023 Sep 23;13(10):1428. doi: 10.3390/jpm13101428 (PMC10607978; doi:10.3390/jpm13101428)
Supplement: Supplementary file 1 [file jpm-13-01428-s001.zip › File S3_quality_appraisal.pdf]

Checklist criterion	Routley, et al.	Rosenau, et al.	Florman, et al.	van Vilsteren, et al.	Marin, et al.
Is the hypothesis/aim/objective of the study stated clearly in the abstract, introduction, or methods section?	*Yes*<br><br>Describes aim to present centre's experience	Yes<br><br>Clear description of aims (survival and role of prognostic indicators)	Yes<br><br>Clearly described	Yes	Yes
Are the characteristics of the participants included in the study described?	*Yes*<br>Adequate description of sex, age, hormonal status, stage and status	Yes	Yes	Yes	No
Were the cases collected in more than one centre?	No<br>Single centre study (Kings College London)	No<br>Single centre study	No<br>Single centre study	No<br>Single centre study	No<br>Single centre study
Are the eligibility criteria (inclusion and exclusion) for entry explicit and appropriate?	No<br>Generic, poorly described selection criteria. Palliative only.	No<br>All patients included, but indications not well described/detailed	No<br>Almost good, but uncertainty around resectability	Yes	No
Were participants recruited consecutively?	Yes	Yes	Yes	Yes	Yes
Did participants enter the study at a similar point in the disease?	No<br>Appear to all be palliative intent, but at different disease stages (i.e. metastases and non-distant metastases; different indications)	No<br>Mixture of localised, nodal, and distantly metastatic disease	No	Yes	Yes<br><br>(all had metastases)
Was the intervention clearly described in the study?	No<br>(minimal to no information on approach, post-op management, selection)	No<br>(minimal detail on selection; standard orthotopic technique stated)	Yes	Yes	No<br>(minimal detail on procedure and selection)
Were additional interventions (co-interventions) clearly reported in the study?	Yes	Yes<br>Excellently described at patient level	Yes	Yes	No<br>(Minimal data provided)
Are the outcome measures clearly defined in the introduction or methods section?	No<br>(No methods section; intro states generic 'outcomes')	Yes	No	Yes	No
Were relevant outcomes appropriately measured with objective and/or subjective methods?	Yes<br>(outcomes studied were relevant, but not exhaustive)	Yes	Yes	Yes	Yes
Were outcomes measured before and after intervention?	Not appropriate<br>(post-surgical outcomes)	Not appropriate	Not appropriate	Not appropriate	Not appropriate

Were the statistical tests used to assess the relevant outcomes appropriate?	Yes	Yes	Yes	Yes	Unclear
Was the length of follow-up reported?	Yes<br>(Albeit, by group, rather than overall)	Yes	Yes	Yes	Yes
Was the loss to follow-up reported?	Yes	Yes	Yes	Yes	Yes
Does the study provide estimates of the random variability in the data analysis of relevant outcomes?	No	No	No	No	No
Are adverse events reported?	Yes	Yes	Yes	Yes	Yes
Are the conclusions of the study supported by results?	Yes	Yes	Yes	Yes	Yes
Are both competing interests and sources of support for the study reported?	No	No	No	No	No
**Total score**	**10**	**11**	**11**	**14**	**9**
Checklist criterion	Olausson, et al.	Dhupar, et al.	Frilling, et al.	Bonaccorsi-Riani, et al.	Le Treut, et al.
Is the hypothesis/aim/objective of the study stated clearly in the abstract, introduction, or methods section?	Yes	Yes	Yes	Yes	Yes
Are the characteristics of the participants included in the study described?	Yes	Yes	No<br>(Yes overall, but not specifically for the transplant sub-set)	Yes	Yes
Were the cases collected in more than one centre?	No	No	No	No	Yes
Are the eligibility criteria (inclusion and exclusion) for entry explicit and appropriate?	Yes	Yes	Yes	Yes	Yes
Were participants recruited consecutively?	Yes	Yes	Yes	Yes	Yes
Did participants enter the study at a similar point in the disease?	Yes<br>(All metastatic stage)	Yes<br>(All metastatic stage)	Yes<br>(All distant metastases, i.e. liver)	Yes<br>(All metastatic stages)	Yes<br>(All metastatic)
Was the intervention clearly described in the study?	Yes<br>(Excellent detail on pre-transplant, and procedural aspects)	No	Yes	Yes	Yes

Were additional interventions (co-interventions) clearly reported in the study?	Yes	No<br>(not specifically for the NET group)	No	Yes<br>(pre-transplant trajectory is detailed)	Yes
Are the outcome measures clearly defined in the introduction or methods section?	Yes	Yes	Yes	No	Yes
Were relevant outcomes appropriately measured with objective and/or subjective methods?	Yes	Yes	Yes	Yes	Yes
Were outcomes measured before and after intervention?	Not appropriate	Not appropriate	Not appropriate	Not appropriate	Not appropriate
Were the statistical tests used to assess the relevant outcomes appropriate?	Yes	Yes	Yes	Yes	Yes<br>(For outcomes; double selection of prognostic factors occurred, i.e. univariate screen then backwards elimination I multivariable Cox model)
Was the length of follow-up reported?	Yes	Yes	Yes	Yes	Yes
Was the loss to follow-up reported?	Yes	Yes	Yes	Yes	Yes
Does the study provide estimates of the random variability in the data analysis of relevant outcomes?	No	No	No	No	No
Are adverse events reported?	Yes	Yes	No	Yes	Yes
Are the conclusions of the study supported by results?	Yes	Yes	Yes	Yes	Yes
Are both competing interests and sources of support for the study reported?	Yes	Yes	Yes	No	Yes
**Total score**	**15**	**13**	**12**	**13**	**16**
Checklist criterion	Sher, et al.	Mazzaferro, et al.	Korda, et al.	Valvi, et al.	Sposito, et al.
Is the hypothesis/aim/objective of the study stated clearly in the abstract, introduction, or methods section?	Yes	Yes	Yes	Yes	Yes
Are the characteristics of the participants included in the study described?	Yes	Yes	No<br>(Limited descriptives)	Yes	Yes

Were the cases collected in more than one centre?	Yes	No	No	Yes	No
Are the eligibility criteria (inclusion and exclusion) for entry explicit and appropriate?	No<br>(Multicentre, no clear data on OLT selection criteria)	Yes<br>(Rare case where explicit selection criteria detailed)	No<br>(Limited detail)	No<br>(Multicentre registry [UNOS], no uniform selection criteria or discussion of differences in centres)	Yes
Were participants recruited consecutively?	No	Yes	Yes	Yes	Yes
Did participants enter the study at a similar point in the disease?	Yes<br>(All metastatic)	Yes	Yes/uncertain	Yes	Yes<br>(All post-liver transplant recurrence)
Was the intervention clearly described in the study?	No<br>(Variability in centres assumed; no unified 'approach' to selection)	Yes	No	Yes	Yes
Were additional interventions (co-interventions) clearly reported in the study?	No	Yes	Yes	No	Yes
Are the outcome measures clearly defined in the introduction or methods section?	Yes	Yes	Yes	Yes	Yes
Were relevant outcomes appropriately measured with objective and/or subjective methods?	Yes	Yes	Yes	Yes	Yes
Were outcomes measured before and after intervention?	Not appropriate	Not appropriate	Not appropriate	Not appropriate	Not appropriate
Were the statistical tests used to assess the relevant outcomes appropriate?	Yes	Yes	Yes	Yes	Yes
Was the length of follow-up reported?	Yes	Yes	Yes	No	Yes<br>(Median follow-up after recurrence was the key aspect, reported at 73.7mo)
Was the loss to follow-up reported?	Yes	Yes	Yes	No	Yes
Does the study provide estimates of the random variability in the data analysis of relevant outcomes?	No	Yes<br>(Accounted for optimism in data analysis when compiling propensity score model using bootstrap and optimism correction; Monte-Carlo	No	No	No

		approaches to confidence intervals).			
Are adverse events reported?	Yes	Yes	Yes	No	No
Are the conclusions of the study supported by results?	Yes	Yes	Yes	Yes	Yes
Are both competing interests and sources of support for the study reported?	Yes	Yes	Yes	Yes	Yes
**Total score**	**12**	**16**	**12**	**11**	**14**
